# (*E*)-3-(2-Chloro-6-methyl-3-quinol­yl)-1-(2,3-dihydro-1,4-benzodioxin-6-yl)prop-2-en-1-one

**DOI:** 10.1107/S1600536810007464

**Published:** 2010-03-03

**Authors:** Syed Umar Farooq Rizvi, Hamid Latif Siddiqui, Tanvir Hussain, Muhammad Azam, Masood Parvez

**Affiliations:** a Institute of Chemistry, University of the Punjab, Lahore 54590, Pakistan; b Institute of Biochemistry, University of Balouchistan, Quetta 7800, Pakistan; c Department of Chemistry, The University of Calgary, 2500 University Drive NW, Calgary, Alberta, Canada T2N 1N4

## Abstract

In the title mol­ecule, C_21_H_16_ClNO_3_, the quinoline and benzene rings are inclined at 56.96 (6)° with respect to each other and the dioxine ring is in a twist-chair conformation. The structure is devoid of any classical hydrogen bonds. Rather weak inter­molecular hydrogen-bonding inter­actions of the types C—H⋯N and C—H⋯O are present, consolidating the crystal structure.

## Related literature

For background to chalcones, see: Mishra *et al.* (2008 ▶); Xia *et al.* (2000 ▶); Vaya *et al.* (1997 ▶); Bhakuni & Chaturvedi (1984 ▶); Nielsen *et al.* (2005 ▶); Wu *et al.* (2003 ▶). For comparison bond lengths, see: Allen *et al.* (1987 ▶). For a related structure, see: Rizvi *et al.* (2010 ▶) For the preparation of the precursor 2-chloro-6-methyl-3-formyl­quinoline, see: Meth-Cohn *et al.* (1981 ▶).
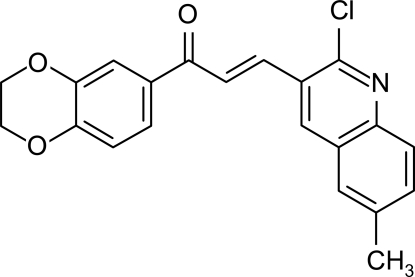


## Experimental

### 

#### Crystal data

C_21_H_16_ClNO_3_
*M* *_r_* = 365.80
Monoclinic, 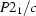

*a* = 6.370 (3) Å
*b* = 38.735 (9) Å
*c* = 7.409 (4) Å
β = 114.93 (2)°
*V* = 1657.8 (12) Å^3^
*Z* = 4
Mo *K*α radiation
μ = 0.25 mm^−1^
*T* = 173 K
0.18 × 0.16 × 0.14 mm

#### Data collection

Nonius KappaCCD diffractometer
Absorption correction: multi-scan (*SORTAV*; Blessing, 1997 ▶) *T*_min_ = 0.956, *T*_max_ = 0.966
6971 measured reflections
2933 independent reflections
2256 reflections with *I* > 2σ(*I*)
*R*_int_ = 0.037

#### Refinement

*R*[*F* ^2^ > 2σ(*F* ^2^)] = 0.037
*wR*(*F* ^2^) = 0.091
*S* = 1.03
2933 reflections
236 parameters
H-atom parameters constrained
Δρ_max_ = 0.19 e Å^−3^
Δρ_min_ = −0.23 e Å^−3^

### 

Data collection: *COLLECT* (Hooft, 1998 ▶); cell refinement: *DENZO* (Otwinowski & Minor, 1997 ▶); data reduction: *SCALEPACK* (Otwinowski & Minor, 1997 ▶); program(s) used to solve structure: *SHELXS97* (Sheldrick, 2008 ▶); program(s) used to refine structure: *SHELXL97* (Sheldrick, 2008 ▶); molecular graphics: *ORTEP-3 for Windows* (Farrugia, 1997 ▶); software used to prepare material for publication: *SHELXL97*.

## Supplementary Material

Crystal structure: contains datablocks global, I. DOI: 10.1107/S1600536810007464/si2245sup1.cif

Structure factors: contains datablocks I. DOI: 10.1107/S1600536810007464/si2245Isup2.hkl

Additional supplementary materials:  crystallographic information; 3D view; checkCIF report

## Figures and Tables

**Table 1:** Hydrogen-bond geometry (Å, °)

*D*—H⋯*A*	*D*—H	H⋯*A*	*D*⋯*A*	*D*—H⋯*A*
C2—H2⋯N1^i^	0.95	2.57	3.514 (3)	170
C18—H18⋯O2^ii^	0.95	2.53	3.266 (3)	134
C21—H21*A*⋯O1^iii^	0.99	2.53	3.406 (3)	147

Symmetry codes: (i) 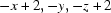; (ii) 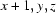; (iii) 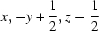.

**Fig. 1.**
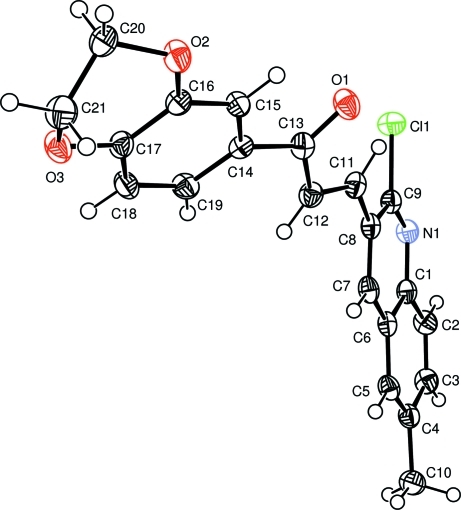
ORTEP-3 (Farrugia, 1997) drawing of (I) with displacement ellipsoids plotted at 50% probability level.

## supplementary crystallographic information

### Comment

α,β-Unsaturated ketones or 1,3-diaryl-2-propen-1-ones, commonly known as chalcones, have already been recognized as antimalarial (Mishra *et al.*, 2008), antitumor (Xia *et al.*, 2000), antioxidant (Vaya *et al.*, 1997), antifungal (Bhakuni & Chaturvedi, 1984), antibacterial (Nielsen *et al.*, 2005), and anti-AIDS agents (Wu *et al.*, 2003). Continuing our investigations in this important area (Rizvi *et al.*, 2010) we now report the synthesis and crystal structure of a new chalcone, containing quinolyl ring system, (2E)-3-(2-chloro-6-methylquinolin-3-yl)-1-(2,3-dihydro-1,4-benzodioxin-6-yl) prop-2-en-1-one, in this article. A series of chalcones related to the title compound is under investigation for their biological activities in our laboratory.

The title molecule is presented in Fig. 1. The bond distances (Allen *et al.*,  1987) and angles are as expected and agree with the corresponding bond distances and angles reported in a closely related compound (Rizvi *et al.*, 2010). The least-square planes of the quinoline and phenyl rings defined by atoms N1/C1—C9 and C14—C19, respectively, are inclined at 56.95 (6)° with respect to each other. The dioxine ring is in a twist-chair conformation with C20 and C21 atoms 0.425 (3) and 0.307 (3) Å on the opposite sides of the plane formed by the remining ring atoms. The structure is devoid of any classical hydrogen bonds. However, short intramolecular interactions involving Cl1 and O1 and rather weak hydrogen bonding inter-molecular interactions of the types C—H···N and C—H···O are present consolidating the crystal packing; details have been provided in Table 1.

### Experimental

The precursor 2-chloro-6-methyl-3-formylquinoline was prepared by reported method (Meth-Cohn *et al.*, 1981). A mixture of 2-chloro-6-methyl-3-formylquinoline (2.055 g, 10 mmol) and 6-acetyl-1,4-benzodioxane (1.7819 g, 10 mmol) in methanol (50 ml) was stirred at room temperature followed by dropwise addition of aq. NaOH (4 ml, 10%). The stirring was continued (2 h) and the reaction mixture was kept at 273 K (24 h). Then it was poured on to ice-cold water (200 ml). The precipitates were collected by filtration, washed with cold water followed by cold MeOH. The resulting chalcone was recrystallised from CHCl_3_ to obtain the title compound as yellow crystalline product, (yield 2.76 g, 7.55 mmol, 75.5%), (m.p. 458-460 K).

### Refinement

Though all the H atoms could be distinguished in the difference Fourier map the H-atoms were included at geometrically idealized positions and refined in riding-model approximation with the following constraints: C—H distances were set to 0.95, 0.98 and 0.99 Å for aromatic, methyl and methylene H-atoms, respectively, and *U*_iso_(H) = 1.2*U*_eq_(C). The final difference map was essentially featurless.

### Crystal data

**Table:**

C_21_H_16_ClNO_3_	*F*(000) = 760
*M**_r_* = 365.80	*D*_x_ = 1.466 Mg m^−^^3^
Monoclinic, *P*2_1_/*c*	Mo *K*α radiation, λ = 0.71073 Å
Hall symbol: -P 2ybc	Cell parameters from 6971 reflections
*a* = 6.370 (3) Å	θ = 3.6–25.3°
*b* = 38.735 (9) Å	µ = 0.25 mm^−^^1^
*c* = 7.409 (4) Å	*T* = 173 K
β = 114.93 (2)°	Prism, colorless
*V* = 1657.8 (12) Å^3^	0.18 × 0.16 × 0.14 mm
*Z* = 4	

### Data collection

**Table:**

Nonius KappaCCD diffractometer	2933 independent reflections
Radiation source: fine-focus sealed tube	2256 reflections with *I* > 2σ(*I*)
graphite	*R*_int_ = 0.037
ω and φ scans	θ_max_ = 25.4°, θ_min_ = 3.6°
Absorption correction: multi-scan (*SORTAV*; Blessing, 1997)	*h* = −7→7
*T*_min_ = 0.956, *T*_max_ = 0.966	*k* = −46→45
6971 measured reflections	*l* = −8→8

### Refinement

**Table:**

Refinement on *F*^2^	Primary atom site location: structure-invariant direct methods
Least-squares matrix: full	Secondary atom site location: difference Fourier map
*R*[*F*^2^ > 2σ(*F*^2^)] = 0.037	Hydrogen site location: difference Fourier map
*wR*(*F*^2^) = 0.091	H-atom parameters constrained
*S* = 1.03	*w* = 1/[σ^2^(*F*_o_^2^) + (0.0323*P*)^2^ + 0.8409*P*] where *P* = (*F*_o_^2^ + 2*F*_c_^2^)/3
2933 reflections	(Δ/σ)_max_ = 0.001
236 parameters	Δρ_max_ = 0.19 e Å^−^^3^
0 restraints	Δρ_min_ = −0.23 e Å^−^^3^

### Special details

**Table:**

Geometry. All esds (except the esd in the dihedral angle between two l.s. planes) are estimated using the full covariance matrix. The cell esds are taken into account individually in the estimation of esds in distances, angles and torsion angles; correlations between esds in cell parameters are only used when they are defined by crystal symmetry. An approximate (isotropic) treatment of cell esds is used for estimating esds involving l.s. planes.
Refinement. Refinement of *F*^2^ against ALL reflections. The weighted *R*-factor wR and goodness of fit *S* are based on *F*^2^, conventional *R*-factors *R* are based on *F*, with *F* set to zero for negative *F*^2^. The threshold expression of *F*^2^ > σ(*F*^2^) is used only for calculating *R*-factors(gt) etc. and is not relevant to the choice of reflections for refinement. *R*-factors based on *F*^2^ are statistically about twice as large as those based on *F*, and *R*- factors based on ALL data will be even larger.

### Fractional atomic coordinates and isotropic or equivalent isotropic displacement parameters (Å^2^)

**Table:**

	*x*	*y*	*z*	*U*_iso_*/*U*_eq_	
Cl1	0.61049 (9)	0.077424 (13)	1.07582 (8)	0.03394 (16)	
O1	0.1834 (2)	0.18022 (3)	0.7158 (2)	0.0336 (4)	
O2	0.0375 (2)	0.30663 (3)	0.6559 (2)	0.0320 (4)	
O3	0.4801 (2)	0.33735 (3)	0.7522 (2)	0.0338 (4)	
N1	0.8742 (3)	0.05188 (4)	0.9228 (2)	0.0267 (4)	
C1	0.9871 (3)	0.05159 (5)	0.8010 (3)	0.0246 (4)	
C2	1.1233 (3)	0.02252 (5)	0.8050 (3)	0.0287 (5)	
H2	1.1381	0.0039	0.8932	0.034*	
C3	1.2333 (3)	0.02116 (5)	0.6821 (3)	0.0306 (5)	
H3	1.3235	0.0014	0.6859	0.037*	
C4	1.2168 (3)	0.04847 (5)	0.5487 (3)	0.0276 (5)	
C5	1.0885 (3)	0.07697 (5)	0.5472 (3)	0.0269 (4)	
H5	1.0790	0.0956	0.4605	0.032*	
C6	0.9699 (3)	0.07956 (5)	0.6704 (3)	0.0243 (4)	
C7	0.8272 (3)	0.10775 (5)	0.6683 (3)	0.0258 (4)	
H7	0.8131	0.1269	0.5838	0.031*	
C8	0.7085 (3)	0.10785 (5)	0.7868 (3)	0.0241 (4)	
C9	0.7448 (3)	0.07847 (5)	0.9125 (3)	0.0255 (4)	
C10	1.3379 (4)	0.04523 (6)	0.4128 (3)	0.0350 (5)	
H10A	1.3123	0.0663	0.3328	0.042*	
H10B	1.2757	0.0253	0.3246	0.042*	
H10C	1.5042	0.0420	0.4928	0.042*	
C11	0.5459 (3)	0.13523 (5)	0.7802 (3)	0.0261 (5)	
H11	0.4158	0.1282	0.8025	0.031*	
C12	0.5602 (3)	0.16871 (5)	0.7467 (3)	0.0268 (5)	
H12	0.6904	0.1774	0.7302	0.032*	
C13	0.3705 (3)	0.19247 (5)	0.7352 (3)	0.0259 (4)	
C14	0.4061 (3)	0.23038 (5)	0.7428 (3)	0.0222 (4)	
C15	0.2138 (3)	0.25160 (5)	0.6999 (3)	0.0239 (4)	
H15	0.0667	0.2415	0.6683	0.029*	
C16	0.2342 (3)	0.28706 (5)	0.7026 (3)	0.0235 (4)	
C17	0.4504 (3)	0.30212 (5)	0.7508 (3)	0.0252 (4)	
C18	0.6434 (3)	0.28131 (5)	0.8000 (3)	0.0300 (5)	
H18	0.7915	0.2916	0.8382	0.036*	
C19	0.6226 (3)	0.24564 (5)	0.7942 (3)	0.0278 (5)	
H19	0.7555	0.2316	0.8250	0.033*	
C20	0.0868 (3)	0.34184 (5)	0.7204 (3)	0.0290 (5)	
H20A	0.1447	0.3430	0.8670	0.035*	
H20B	−0.0566	0.3558	0.6607	0.035*	
C21	0.2653 (4)	0.35620 (5)	0.6590 (3)	0.0346 (5)	
H21A	0.2077	0.3547	0.5125	0.041*	
H21B	0.2925	0.3808	0.6974	0.041*	

### Atomic displacement parameters (Å^2^)

**Table:**

	*U*^11^	*U*^22^	*U*^33^	*U*^12^	*U*^13^	*U*^23^
Cl1	0.0392 (3)	0.0327 (3)	0.0351 (3)	0.0045 (2)	0.0207 (2)	0.0033 (2)
O1	0.0288 (8)	0.0239 (8)	0.0471 (9)	−0.0005 (6)	0.0150 (7)	0.0008 (6)
O2	0.0259 (7)	0.0194 (7)	0.0490 (9)	0.0032 (6)	0.0141 (7)	−0.0016 (6)
O3	0.0339 (8)	0.0204 (7)	0.0498 (9)	−0.0021 (6)	0.0202 (7)	0.0013 (7)
N1	0.0287 (9)	0.0218 (9)	0.0289 (9)	0.0011 (7)	0.0113 (8)	0.0026 (7)
C1	0.0238 (10)	0.0200 (10)	0.0269 (11)	−0.0003 (8)	0.0077 (9)	−0.0001 (8)
C2	0.0314 (11)	0.0216 (10)	0.0333 (12)	0.0026 (8)	0.0138 (9)	0.0055 (9)
C3	0.0312 (11)	0.0228 (11)	0.0375 (12)	0.0034 (9)	0.0142 (10)	0.0004 (9)
C4	0.0267 (10)	0.0237 (11)	0.0323 (11)	−0.0023 (8)	0.0123 (9)	−0.0019 (9)
C5	0.0281 (10)	0.0220 (10)	0.0291 (11)	−0.0034 (8)	0.0106 (9)	0.0039 (8)
C6	0.0213 (9)	0.0199 (10)	0.0278 (11)	−0.0024 (8)	0.0066 (8)	−0.0018 (8)
C7	0.0270 (10)	0.0175 (10)	0.0289 (11)	−0.0025 (8)	0.0078 (9)	0.0030 (8)
C8	0.0232 (10)	0.0185 (10)	0.0263 (10)	−0.0018 (8)	0.0063 (8)	−0.0011 (8)
C9	0.0267 (10)	0.0224 (10)	0.0264 (10)	−0.0025 (8)	0.0102 (8)	−0.0015 (8)
C10	0.0375 (12)	0.0321 (12)	0.0404 (13)	−0.0001 (9)	0.0214 (11)	0.0003 (10)
C11	0.0246 (10)	0.0220 (11)	0.0278 (11)	0.0003 (8)	0.0072 (9)	−0.0027 (8)
C12	0.0265 (10)	0.0247 (11)	0.0282 (11)	0.0016 (8)	0.0104 (9)	−0.0012 (8)
C13	0.0267 (10)	0.0246 (11)	0.0234 (10)	−0.0003 (8)	0.0075 (9)	0.0014 (8)
C14	0.0258 (10)	0.0209 (10)	0.0206 (10)	0.0025 (8)	0.0103 (8)	0.0010 (8)
C15	0.0222 (10)	0.0228 (11)	0.0265 (10)	−0.0013 (8)	0.0101 (8)	0.0002 (8)
C16	0.0249 (10)	0.0219 (10)	0.0233 (10)	0.0024 (8)	0.0097 (8)	0.0006 (8)
C17	0.0312 (11)	0.0183 (10)	0.0271 (11)	−0.0010 (8)	0.0131 (9)	−0.0003 (8)
C18	0.0246 (10)	0.0279 (11)	0.0385 (12)	−0.0055 (9)	0.0144 (9)	−0.0022 (9)
C19	0.0234 (10)	0.0270 (11)	0.0341 (11)	0.0041 (8)	0.0130 (9)	0.0015 (9)
C20	0.0336 (11)	0.0204 (11)	0.0328 (11)	0.0040 (9)	0.0137 (9)	−0.0005 (9)
C21	0.0417 (13)	0.0219 (11)	0.0426 (13)	0.0055 (9)	0.0202 (11)	0.0044 (9)

### Geometric parameters (Å, °)

**Table:**

Cl1—C9	1.752 (2)	C10—H10A	0.9800
O1—C13	1.234 (2)	C10—H10B	0.9800
O2—C16	1.378 (2)	C10—H10C	0.9800
O2—C20	1.435 (2)	C11—C12	1.331 (3)
O3—C17	1.377 (2)	C11—H11	0.9500
O3—C21	1.444 (2)	C12—C13	1.492 (3)
N1—C9	1.301 (2)	C12—H12	0.9500
N1—C1	1.371 (3)	C13—C14	1.483 (3)
C1—C2	1.414 (3)	C14—C15	1.396 (3)
C1—C6	1.426 (3)	C14—C19	1.398 (3)
C2—C3	1.364 (3)	C15—C16	1.379 (3)
C2—H2	0.9500	C15—H15	0.9500
C3—C4	1.421 (3)	C16—C17	1.396 (3)
C3—H3	0.9500	C17—C18	1.385 (3)
C4—C5	1.371 (3)	C18—C19	1.387 (3)
C4—C10	1.509 (3)	C18—H18	0.9500
C5—C6	1.413 (3)	C19—H19	0.9500
C5—H5	0.9500	C20—C21	1.498 (3)
C6—C7	1.416 (3)	C20—H20A	0.9900
C7—C8	1.379 (3)	C20—H20B	0.9900
C7—H7	0.9500	C21—H21A	0.9900
C8—C9	1.426 (3)	C21—H21B	0.9900
C8—C11	1.469 (3)		
			
C16—O2—C20	112.99 (15)	C8—C11—H11	116.3
C17—O3—C21	113.44 (15)	C11—C12—C13	119.61 (19)
C9—N1—C1	117.57 (16)	C11—C12—H12	120.2
N1—C1—C2	118.71 (17)	C13—C12—H12	120.2
N1—C1—C6	121.93 (17)	O1—C13—C14	120.65 (18)
C2—C1—C6	119.36 (18)	O1—C13—C12	119.25 (18)
C3—C2—C1	120.01 (18)	C14—C13—C12	120.09 (18)
C3—C2—H2	120.0	C15—C14—C19	118.90 (18)
C1—C2—H2	120.0	C15—C14—C13	117.97 (17)
C2—C3—C4	121.82 (19)	C19—C14—C13	123.12 (17)
C2—C3—H3	119.1	C16—C15—C14	121.01 (18)
C4—C3—H3	119.1	C16—C15—H15	119.5
C5—C4—C3	118.31 (19)	C14—C15—H15	119.5
C5—C4—C10	122.13 (18)	O2—C16—C15	118.31 (17)
C3—C4—C10	119.56 (18)	O2—C16—C17	121.94 (17)
C4—C5—C6	122.10 (18)	C15—C16—C17	119.75 (17)
C4—C5—H5	119.0	O3—C17—C18	118.09 (18)
C6—C5—H5	119.0	O3—C17—C16	122.24 (17)
C5—C6—C7	124.26 (18)	C18—C17—C16	119.67 (18)
C5—C6—C1	118.38 (17)	C17—C18—C19	120.62 (19)
C7—C6—C1	117.32 (18)	C17—C18—H18	119.7
C8—C7—C6	121.09 (17)	C19—C18—H18	119.7
C8—C7—H7	119.5	C18—C19—C14	120.00 (18)
C6—C7—H7	119.5	C18—C19—H19	120.0
C7—C8—C9	115.58 (17)	C14—C19—H19	120.0
C7—C8—C11	123.31 (17)	O2—C20—C21	109.83 (16)
C9—C8—C11	121.05 (18)	O2—C20—H20A	109.7
N1—C9—C8	126.48 (19)	C21—C20—H20A	109.7
N1—C9—Cl1	114.99 (15)	O2—C20—H20B	109.7
C8—C9—Cl1	118.51 (15)	C21—C20—H20B	109.7
C4—C10—H10A	109.5	H20A—C20—H20B	108.2
C4—C10—H10B	109.5	O3—C21—C20	110.74 (17)
H10A—C10—H10B	109.5	O3—C21—H21A	109.5
C4—C10—H10C	109.5	C20—C21—H21A	109.5
H10A—C10—H10C	109.5	O3—C21—H21B	109.5
H10B—C10—H10C	109.5	C20—C21—H21B	109.5
C12—C11—C8	127.38 (19)	H21A—C21—H21B	108.1
C12—C11—H11	116.3		
			
C9—N1—C1—C2	−178.11 (17)	C8—C11—C12—C13	176.79 (18)
C9—N1—C1—C6	1.5 (3)	C11—C12—C13—O1	−14.8 (3)
N1—C1—C2—C3	178.47 (18)	C11—C12—C13—C14	166.44 (18)
C6—C1—C2—C3	−1.1 (3)	O1—C13—C14—C15	−8.8 (3)
C1—C2—C3—C4	0.3 (3)	C12—C13—C14—C15	169.95 (17)
C2—C3—C4—C5	0.9 (3)	O1—C13—C14—C19	170.08 (19)
C2—C3—C4—C10	−178.64 (19)	C12—C13—C14—C19	−11.2 (3)
C3—C4—C5—C6	−1.3 (3)	C19—C14—C15—C16	1.7 (3)
C10—C4—C5—C6	178.19 (18)	C13—C14—C15—C16	−179.44 (17)
C4—C5—C6—C7	−177.15 (18)	C20—O2—C16—C15	162.26 (17)
C4—C5—C6—C1	0.5 (3)	C20—O2—C16—C17	−18.1 (3)
N1—C1—C6—C5	−178.89 (17)	C14—C15—C16—O2	179.02 (17)
C2—C1—C6—C5	0.7 (3)	C14—C15—C16—C17	−0.6 (3)
N1—C1—C6—C7	−1.0 (3)	C21—O3—C17—C18	167.52 (18)
C2—C1—C6—C7	178.55 (17)	C21—O3—C17—C16	−12.7 (3)
C5—C6—C7—C8	177.13 (18)	O2—C16—C17—O3	−0.9 (3)
C1—C6—C7—C8	−0.6 (3)	C15—C16—C17—O3	178.76 (17)
C6—C7—C8—C9	1.6 (3)	O2—C16—C17—C18	178.86 (18)
C6—C7—C8—C11	−175.60 (17)	C15—C16—C17—C18	−1.5 (3)
C1—N1—C9—C8	−0.4 (3)	O3—C17—C18—C19	−177.65 (18)
C1—N1—C9—Cl1	−178.80 (13)	C16—C17—C18—C19	2.6 (3)
C7—C8—C9—N1	−1.2 (3)	C17—C18—C19—C14	−1.6 (3)
C11—C8—C9—N1	176.08 (18)	C15—C14—C19—C18	−0.6 (3)
C7—C8—C9—Cl1	177.23 (14)	C13—C14—C19—C18	−179.41 (18)
C11—C8—C9—Cl1	−5.5 (2)	C16—O2—C20—C21	48.1 (2)
C7—C8—C11—C12	−34.3 (3)	C17—O3—C21—C20	43.1 (2)
C9—C8—C11—C12	148.6 (2)	O2—C20—C21—O3	−62.2 (2)

### Hydrogen-bond geometry (Å, °)

**Table:**

*D*—H···*A*	*D*—H	H···*A*	*D*···*A*	*D*—H···*A*
C11—H11···Cl1	0.95	2.72	3.036 (2)	100
C11—H11···O1	0.95	2.42	2.769 (2)	101
C2—H2···N1^i^	0.95	2.57	3.514 (3)	170
C18—H18···O2^ii^	0.95	2.53	3.266 (3)	134
C21—H21A···O1^iii^	0.99	2.53	3.406 (3)	147

Symmetry codes: (i) −*x*+2, −*y*, −*z*+2; (ii) *x*+1, *y*, *z*; (iii) *x*, −*y*+1/2, *z*−1/2.
